# HIV drug resistance in children with treatment failure to first-line regimens in Ho Chi Minh City, Vietnam

**DOI:** 10.1186/1758-2652-13-S4-P140

**Published:** 2010-11-08

**Authors:** VTT Nhung, D Colby, TH Khanh, TT Viet, D Lu, HT Thuy, B An, LT Giang

**Affiliations:** 1 Tropical Diseases Hospital, HAIVN Project, Ho Chi Minh, Vietnam; 2 Pediatric Hospital Number 1, Ho Chi Minh, Vietnam; 3 Pediatric Hospital Number 2, Ho Chi Minh, Vietnam; 4 Provincial AIDS Committee, Ho Chi Minh, Vietnam

## Background

ARV resistance in children with first-line ARV treatment failure in Vietnam is unknown because antiretroviral therapy (ART) has been available on a large scale only since Sept 2005 and resistance testing is not widely performed. We characterize patterns of mutations to identify common resistance patterns for choosing the second-line regimens in Ho Chi Minh City.

## Methods

54 HIV-infected children were identified as having suspected treatment failure from June to December 2008 in HCMC. Selection criteria were one of the clinical or immunological criteria defined by the Vietnam Ministry of Health (development or recurrence of a WHO stage III or IV condition, failure to thrive, slow growth, CD4 falling below pretreatment value or below 50% of peak treatment value). The genotypic test was done at Pasteur Institute (RT-PCR Promega, DTCS Beckman Coulter) and analyzed using Stanford University's HIV drug resistance database.

## Results

Most of children were on first line regimens: 17 (31.4%) on D4T+3TC+NVP, 7 (13%) on D4T+3TC+EFV, 13 (24.1%) on AZT+3TC+NVP, and 8 (14.8%) on AZT+3TC+EFV. Six patients were on NRTI-only regimens and 3 patients were on PI-containing regimens due to allergy or intolerance to NNRTI. Mean duration of treatment was 15 months. Viral load was undetectable in 14 samples (26.9%). Genotype tests results were available on 36 patients. Overall, one mutation was detected in 34 (94.4%) patients. NRTI mutations were found in 33 (91.7%), NNRTI mutations in 25 (69.4%), and PI mutations in 3 (8.3%). Among the NRTI mutations, at least one Thymidine Analog Mutation (TAM) defined as M41L, D67N, K70R, L210W, T215Y/F, and K219Q/E, was found in 24 patients (72.7%), of which 17 (70.8%) patients had >3 TAMs. M184V was detected in 22 (66.7%); K65R in 3 (9.1%). Combinations of TAMs+M184V and TAMs+K65R mutations were present in 15 (45.4%) and 2 (6.1%), respectively. For NNRTI mutations, Y181C was most frequent 14 (56%), followed by G190A/S 11 (44%), K101K 7 (28%), K103N 4 (16), V108I 3 (12%) and Y188L (8%). Only three patients with one single PI mutation (33.3%) including M46I, L10F and A71V were found.

## Conclusions

TAMs and the M184V mutation were present in a majority of genotypic tests. K65R and Q151M mutations were less common. The second-line regimen TDF+3TC+LPV/r would be more effective than ABC+DDI+LPV/r for most children with virological failure on first-line ARV in HCMC. More options for second-line in developing countries are needed.

